# Multigene manipulation of photosynthetic carbon metabolism enhances the photosynthetic capacity and biomass yield of cucumber under low-CO_2_ environment

**DOI:** 10.3389/fpls.2022.1005261

**Published:** 2022-10-18

**Authors:** Zhi-Feng Chen, Tian-Hong Wang, Chao-Yang Feng, Hai-Feng Guo, Xiao-Xi Guan, Tian-Li Zhang, Wen-Zhao Li, Guo-Ming Xing, Sheng Sun, Guo-Fei Tan

**Affiliations:** ^1^ College of Biology and Agricultural Technology, Zunyi Normal College, Zunyi, China; ^2^ Fruit and Vegetable Research Institute, Academy of Agricultural Sciences, Zunyi, China; ^3^ College of Horticulture, Shanxi Agricultural University, Jinzhong, China; ^4^ Institute of Horticulture, Guizhou Academy of Agricultural Sciences, Guiyang, China

**Keywords:** cucumber, carbon metabolism, low-CO_2_, greenhouse, multigene manipulation, highphotosynthetic efficiency

## Abstract

Solar greenhouses are important in the vegetable production and widely used for the counter-season production in the world. However, the CO_2_ consumed by crops for photosynthesis after sunrise is not supplemented and becomes chronically deficient due to the airtight structure of solar greenhouses. Vegetable crops cannot effectively utilize light resources under low-CO_2_ environment, and this incapability results in reduced photosynthetic efficiency and crop yield. We used cucumber as a model plant and generated several sets of transgenic cucumber plants overexpressing individual genes, including *β-carbonic anhydrase 1* (*CsβCA1*), *β-carbonic anhydrase 4* (*CsβCA4*), and *sedoheptulose-1,7-bisphosphatase* (*CsSBP*); *fructose-1,6-bisphosphate aldolase* (*CsFBA*), and *CsβCA1* co-expressing plants; *CsβCA4*, *CsSBP*, and *CsFBA* co-expressing plants (*14SF*). The results showed that the overexpression of *CsβCA1*, *CsβCA4*, *and 14SF* exhibited higher photosynthetic and biomass yield in transgenic cucumber plants under low-CO_2_ environment. Further enhancements in photosynthesis and biomass yield were observed in *14SF* transgenic plants under low-CO_2_ environment. The net photosynthesis biomass yield and photosynthetic rate increased by 49% and 79% compared with those of the WT. However, the transgenic cucumbers of overexpressing *CsFBA* and *CsSBP* showed insignificant differences in photosynthesis and biomass yield compared with the WT under low-CO_2_.environment. Photosynthesis, fluorescence parameters, and enzymatic measurements indicated that *CsβCA1*, *CsβCA4*, *CsSBP*, and *CsFBA* had cumulative effects in photosynthetic carbon assimilation under low-CO_2_ environment. Co-expression of this four genes (*CsβCA1*, *CsβCA4*, *CsSBP*, and *CsFBA*) can increase the carboxylation activity of RuBisCO and promote the regeneration of RuBP. As a result, the *14SF* transgenic plants showed a higher net photosynthetic rate and biomass yield even under low-CO_2_environment.These findings demonstrate the possibility of cultivating crops with high photosynthetic efficiency by manipulating genes involved in the photosynthetic carbon assimilation metabolic pathway.

## Introduction

The Calvin cycle is the primary pathway for photosynthetic carbon fixation in higher plants. It consists of three major phases: carboxylation of ribulose-1,5-bisphosphate (RuBP), reduction of 3-phosphoglycerate, and regeneration of the carbon dioxide (CO_2_) acceptor RuBP. In the RuBP reaction, carbonic anhydrase (CA) proteins catalyze the conversion of CO_2_ to bicarbonate ion and protons with zinc iron (Rudenko et al., 2021). CAs are ubiquitous, and higher plants contain three evolutionarily distinct CA families, with βCA being the most predominant (DiMario et al., 2017). In *Arabidopsis*, eight *αCA* and six *βCA* genes have been isolated and characterized, and the correct subcellular localizations of these coding proteins are essential for efficient physiological functioning (Shen et al., 2021). The well-characterized *AtβCA1*, which is located in the chloroplasts, mainly contributes to converting HCO_3_^−^ into CO_2_, which is the substrate for RuBP (Dąbrowska-Bronk et al., 2016). *βCA*-mediated functions are the upstream factor controlling CO_2_ triggered stomata movements, and *βCA* overexpressing plants exhibit instantaneously enhanced water-use efficiency, which indicates the key role of CAs in CO_2_ influx in plant photosynthesis. Further analysis also has proven the potential regulatory role of *βCAs* for optimal plant growth by changing their expression and enzymatic activities (Dąbrowska-Bronk et al., 2016). Cytosolic βCAs can provide bicarbonate for phosphoenolpyruvate carboxylase for C_4_ photosynthesis or for CO_2_ fixation in C_3_ plants (DiMario et al., 2017). To date, cDNAs encoding βCA isoforms have been isolated and identified in a wide range of plant species. *βCA* mutants with altered *βCA* gene expression in many model species, such as *Arabidopsis* (Perales et al., 2005), *Medicago* (López-Millán et al., 2005), *Oryza sativa* (Chen et al., 2017), *Zea mays* (Studer et al., 2014), and *Solanum lycopersicum* (Diamantopoulos et al., 2013), have confirmed that *βCAs* are critical for photosynthesis. These results have indicated that increasing the activity of βCA enzymes could be part of the solution to improving the photosynthetic capacities of plants.

The photosynthesis rate in the C_3_ cycle is also limited by RuBP regeneration, which is determined by the capacity of the electron transport chain to supply adenosine triphosphate and nicotinamide adenine dinucleotide phosphate for supporting the regenerative reactions of the Calvin cycle. Eight enzymes are involved in this process (Zhu et al., 2010; Evans, 2013; Li et al., 2020). Kinetic models based on ordinary differential equations have been developed to describe the responses of photosynthetic carbon assimilation, and this modeling work suggested that the increase in SBP, FBA, and ADP-glucose pyrophosphorylase (AGPase), as well as the decrease in the photorespiratory enzyme glycine decarboxylase (GDC), could increase photosynthetic carbon assimilation (Simkin et al., 2015). In addition to these theoretical predictions, the transgenetic studies in recent years have shown that *SBP* and *FBA* are the major control points of carbon assimilation. The characterization of individual enzymes using RNA interference to manipulate their transcript levels showed that the small reductions in SBP and FBA have obvious limitations on photosynthetic carbon assimilation and that these enzymes had significant control over the rate of carbon assimilation (Bi et al., 2017). Studies in tobacco showed that plants overexpressing *SBP* exhibited an increase in photosynthesis, and leaf area and total biomass was up by as much as 30% in plants grown in high light (Lefebvre et al., 2005). *FBA* overexpression in tobacco significantly enhances plant growth, simulates RuBP regeneration, and promotes CO_2_ fixation (Uematsu et al., 2012). These experiments suggested that improvements in photosynthetic carbon fixation may be achieved by engineering the key genes individually in C_3_ cycle. Moreover, SBP and FBA can be the candidate targets of genetic engineering for improving photosynthetic carbon fixation. However, the above mentioned studies have focused mainly on the function of photosynthetic carbon-fixed genes under normal atmospheric conditions or high-concentration CO_2_ environments. Rosenthal et al. (2011) generated SBP overexpressing transgenic tobacco, and founding that the transgenic plants showed only a minimal increase in greenhouse conditions in winter, while the photosynthetic and biomass yields of the transgenic plants were significantly improved under an elevated CO_2_ enrichment (Rosenthal et al., 2011). Similarly, the overexpression of *FBA* in transgenic tobacco plants resulted in increased photosynthesis and biomass, but this effect was only significant in elevated CO_2_ condition (Uematsu et al., 2012). Few reports are available on the use of genetic engineering to improve the photosynthesis and biomass of crops under low-CO_2_ environments.

Solar greenhouse is one of most important production facilities in the world. Under this situation, increasing the CO_2_ usage efficiency in the greenhouse atmosphere has been proven to improve the productivity of greenhouse crops (Pan et al., 2020). Cucumber is one of the most popular greenhouse crops in the world. The CO_2_ compensation point of a single cucumber leaf is 50~70 µmol·mol^−1^ (Chen et al., 2019). Given the large area coefficient and strong photosynthetic capacity of cucumber leaves, the CO_2_ concentration of cucumber populations is 10% lower than that of the external environment when all of the greenhouse vents are open in the summer. If the vents are completely closed, then the CO_2_ concentration will drop rapidly to 50~100 µmol·mol^−1^ (**Supplementary File S1, Figure S1**), leading to cucumber photosynthesis stops. Improving the photosynthetic capacity under low-CO_2_ concentrations in solar greenhouses remains an important approach for ensuring high greenhouse crop yields.

Co-expression of multiple genes is an effective method to achieve synchronous expression of multigene in a single recipient plant. Previous studies have shown that the introduction of the *inorganic CO_2_ transporter B* gene (involved in carbon transport and carbon concentrating mechanism in *Cyanobacteria*) together with the overexpression of SBP and FBA in transgenic tobacco resulted in an increase in photosynthesis and biomass (Simkin et al., 2015). In our previous study, we introduced the pathway of exogenous glycolate catabolic into cucumber and the transgenic plants showed higher photosynthetic efficiency and biomass yield even in a low-concentration CO_2_ environment (Chen et al., 2019). We aimed to test the hypothesis that gene-stacking of enzyme genes involved in C3 cycle and carbon concentrating mechanism have a synergistic effect on photosynthesis and biomass yield under low-CO_2_ environment. For this purpose, we generated single genes, including *CsβCA1*, *CsβCA4*, *CsSBP*, and *CsFBA* overexpression transgenic cucumber lines, and four multigene co-overexpression transgenic cucumber plants (*14SF*), to explore the feasibility of multigene manipulation of photosynthetic carbon transport and assimilation for improving the photosynthetic capacity and the biomass yield of cucumber under low-CO_2_ environment. The results indicated the potential for improving the photosynthesis and biomass yield of cucumber plants by modifying the expression of a subset of photosynthesis-related genes under low-CO_2_ greenhouse conditions.

## Materials and methods

### Plant material and growth conditions

The cucumber inbred line R3407 used in this study was provided by W. J. Wang at Shanxi Agriculture University, China. The cucumber seedlings were grown under 16 h/8 h and 25 °C/18 °C day/night to the three-true-leaf stage. Then, the cucumber plants were transferred to a controlled low-CO_2_ illumination incubator for further analysis. Gene expression levels of *CsβCA1*, *CsβCA4*, *CsFBA*, and *CsSBP* were detected in the leaf, flower, and growing tips after the flowering of the cucumber plants.

The T_1_ transgenic cucumber seedlings were cultivated in a controlled low-CO_2_ illumination incubator (Percival ARC-36L2-E,Perry, IA, USA) to determine the effects of *CsβCA1*, *CsβCA4*, *CsFBA*, and *CsSBP* overexpression on the growth of cucumber under low-CO_2_ environment. The CO_2_ concentration in the incubator was set to 150 μmol·mol^−1^.After 28 days, the expression and protein accumulation of *CsβCA1*, *CsβCA4*, *CsFBA*, and *CsSBP* were detected in the third leaves. Growth characteristics, such as plant height, stem diameter, and left area, were measured after 28 days after treatment. Photosynthesis and chlorophyll fluorescence parameters were measured in the third leaves at 35 days after growth under low-CO_2_ environment. Transgenic plants were self-pollinated, which leaves only one fruit per plant. The cucumber of fresh and dry weight was measured at 42 days after growth under low-CO_2_ environment.

### Identification of *CsβCA1*, *CsβCA4*, *CsFBA*, and *CsSBP* in the cucumber genome

The amino acid sequences of well-documented SBP, FBA, and βCA in various species were obtained from the Phytozome v12 (http://phytozome.jgi.doe.gov) or *Arabidopsis* Information Resource (http://www.arabidopsis.org) through BLAST analysis with the sequence of CsβCA, CsSBP, and CsFBA protein in cucumber to identify the SBP, FBA, and βCA family members in cucumber. The sequences were examined manually for apparent completeness and correctness in MEGA7 (Kumar et al., 2016). The following databases were used in the search: *Chlamydomonas reinhardtii*, *Physcomitrella patens*, *Amborella trichopoda*, *B. distachyon*, *O. sativa*, *Sorghum bicolor*, *Aquilegia coerulea*, *Linumu sitatissimum*, *Gossypium raimondii*, *Glycine max*, *Brassica rapa*, and *A. thaliana* from Phytozome v12 (http://phytozome.jgi.doe.gov/pz/portal.html) and *C. sativus* L. var. cv. 9930 from the cucumber genome database (http://cucurbitgenomics.org).

### Phylogenic analysis of βCA, SBP, and FBA among angiosperm plant species

The amino acid sequences of βCA, SBP, and FBA in the examined plant species were separately subjected to multiple alignment using fast Fourier transform (MAFFT) (Jasim et al., 2020) to generate multiple sequence alignments with default parameters for revealing the evolutionary history. ProtTest was used to estimate the best model in this analysis for finding the amino acid substitution model that best fitted our data (Wang et al., 2022). The JTT+ Gamma model was the best-fit model for the βCA and SBP protein datasets, whereas the JTT model was the best amino acid substitution model for the FBA protein dataset. The phylogenetic tree was generated with PhyML by using the maximum likelihood method and best protein evolution model with 1000 bootstrap replications each (Leibman et al., 2018), and the final tree was viewed in MEGA7 software.

### Gene cloning and synthesis

The 2A sequence, which originated from FMDV, was used to construct a polycistronic expression vector in this study (Hatazawa et al., 2021). The coding sequences were synthesized using codon optimized genes for expression in cucumber. To obtain the full length cDNA of the Cs*βCA1*, Cs*βCA4*, Cs*FBA*, and Cs*SBP* genes in cucumber, total RNA was extracted from the cucumber fresh leaves by using a Quick-RNA isolation kit, and cDNA was synthesized using a Promega reverse transcriptase kit (Promega, Madison, WI, USA). The gene coding regions were amplified with the designed primers (**Supplementary File S1, Table S1**). The recovered DNA product was then inserted into pMD-18T vectors (TransGenBiotech, Beijing, China) for gene sequencing.

### Construction of expression vectors

In this study, we constructed five expression vectors, four single gene vectors, and one multigene construct vector. To construct the four separated vectors, we amplified a chloroplast-targeting peptide sequence SSU from *Arabidopsis* (**Supplementary File S1, Table S2**) (Lee et al., 2006), and this SSU was ligated at the 5’-terminal of each gene. The fused cDNA fragments encoding the four separate genes were inserted into pCambia1305.1 to generate overexpression constructs, which were transformed into *Agrobacterium* strain C58C1 for plant transformation. The FlagTag was added to the 3’-terminal of each gene to facilitate the detection of the four expression vectors (Liang and Lindblad, 2017). The SSU target peptide was ligated at the 5’-terminal of each gene to generate a multigene construct consisting of CsβCA1, CsβCA4, CsSBP, and CsFBA in tandem. Then, a synthetic 2A linker peptide from FMDV (**Supplementary File S1, Table S2**) was used to combine CsβCA1 and CsβCA4, whereas CsSBP and CsFBA were combined according to the same strategy. Finally, CsβCA1, CsβCA4, CsSBP, and CsFBA were combined to generate a multigene expression construct by using pCambia1305.1 as the backbone. The multigene construct vector was also combined with the Flag-Tag similar to the four single gene expression vectors described previously.

### Cucumber transformation

The cucumber cultivars were transformed with *Agrobacterium* containing the resultant plasmid using the *Agrobacterium*-mediated method described previously, with slight modifications (Wang et al., 2015).

### Identification of transgenic plants

Total DNA and total leaf protein were extracted using a plant DNA extraction kit (Omega Bio-Tek, USA) and plant protein extract kit (Solarbio, China) to detect whether the transgenic plants were successfully constructed. Transformed cucumber plants were selected by PCR and Western blot analysis. A pair of primers (forward primer from the 35S promoter and reverse primer from the target genes) was used to verify the presence of the target transgene using qRT-PCR. Proteins were separated by SDS-PAGE and analyzed by Western blot analysis by using Mouse Anti-Flag Tag antibody (1:500, Bioss, Beijing, China) against the Flag Tag protein (Primers and antibodies are listed in **Supplementary File S1, Table S1**). The positive transformants were used for further gene expression analysis.

### Gene expression analysis

Total RNAs were extracted from the leaves of confirmed transgenic cucumber plants using TRIzol Reagent (Invitrogen, Carlsbad, CA, USA) for cDNA synthesis. qRT-PCR was conducted in a 20 µL reaction mix using an SYBRP remix *Ex* Taq kit following the instructions of the manufacturer (Transgen, Beijing, China) to detect the expression of *CsβCA1*, *CsβCA4*, *CsSBP*, and *CsFBA* (primers are listed in **Supplementary File S1, Table S1**). The qRT-PCR was performed following Wang et al. (2015).

### Quantification of protein

Equal amounts of fresh leaf tissues (0.1 g) at the same position were harvested from the youngest fully expanded leaves of each transgenic plant and were quickly ground to a fine powder in liquid nitrogen for protein quantification. Protein amounts were detected using horseradish peroxidase conjugated to the secondary antibody for the ELISA assay. The antibody information is provided in **Supplementary Table S1**. The activity of RuBisCO was also measured using ELISA.

### Photosynthesis and fluorescence parameter analysis

Photosynthesis and chlorophyll fluorescence measurements were performed on 35-day cucumber seedlings grown under low-CO_2_ illumination incubatorin which the CO_2_concentration was set to 150 μmol·mol^−1^ (Faus et al., 2015). The third leaves were used to measure the Pn and photosynthetic daily variation curve by using a Li-6400 photosynthetic instrument (Faus et al., 2015). During the measurement, the CO_2_ concentration was maintained at 150µmol·mol^−1^, and the leaf temperature was maintained at 25°C.

We detected the Fv/Fm and YII for the third functional leaves using the MINI-PAM modulation chlorophyll fluorescence instrument to further evaluate the potential changes in photosynthesis (Faus et al., 2015). The measurement was performed after 30 min of dark adaptation (Bi et al., 2017).

### Growth index analysis

After 42 days, we determined the effects of the overexpression of target genes on the growth of cucumber plants under low-CO_2_ environment (the CO_2_ concentration was 150 μmol·mol^−1^) by measuring the plant heights with a ruler, stem diameters with a Vernier caliper, leaf lengths and widths with a ruler, and single fruit weight with an electronic scale in T1 transgenic cucumber plants. After these measurements, the samples were dried and weighed to determine the dry weight of the belowground parts.

## Results

### Identification and characterization of *CsβCA, CsFBA*, and *CsSBP* family genes in cucumber

**Figure 1:**
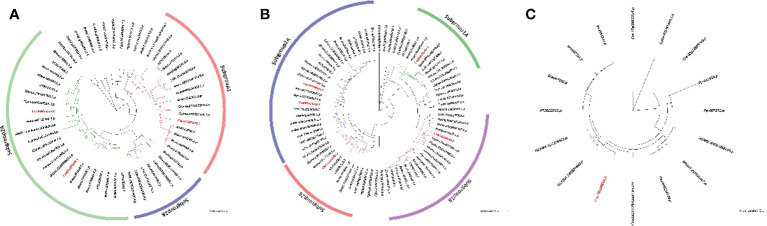
Phylogenetic tree of βCA **(A)**, FBA **(B)** and SBP **(C)** in plant species. Unrooted phylogenetic tree was calculated with the Maximum-Likelihood method, using JTT modeling with gamma-distributed rates and 1000 bootstrap replications based on the multiple sequence alignments of βCA and SBP. The final tree was then rooted in the single clade consisted of members from *C. reinhardtii*. Phylogenetic tree was also constructed with JTT model for FBA proteins and the tree was rooted in the middle of two groups of members from *C. reinhardtii*. Bootstrap values are indicated at the base of each clade. All target protein in cucumber genome were colored with red in those trees.

The full-length alignments of the ProCA, Glycolytic, and FBPase and FBPase_C domains obtained from PFam database were used as queries to search the cucumber proteome database using HMMER software for identifying the *CsFBA*, *CsSBP*, and *CsβCA* family genes in *C. sativus*. We identified three *CsβCAs*, one *CsSBP*, and five *CsFBAs* candidate genes for the *C. sativus* genome (**Table 1**). Three maximum-likelihood phylogenetic trees were constructed based on the full-length alignments of βCAs, SBPs, and FBAs protein sequences from 11 representative plants to further explore the evolutionary relationships of each gene family (**Figure 1**). *βCA* family genes of these plants were further classified into two groups: groups I and II. Groups I and II contained members from monocots and dicots. All monocots contained one copy of the *βCA* genes, and most dicots contained two or more copies, which suggests that gene duplication events may have occurred in the common ancestor of dicots (**Figure 1A**). Phylogenetic analysis of the *FBA* family gene found that the FBAs in land plants could be classified into four groups (I, II, III, and IV). Groups I and IV only contained a single copy of the *FBA* genes, and groups II and III contained multiple copies of the *FBA* genes, which implies that gene duplication events occurred in the common ancestor of seed plants for the *FBA* genes of groups II and III (**Figure 1B**). Moreover, only one copy of the *SBP* genes was identified in most seed plants (**Figure 1C**). Further analysis showed that the *βCA1*, *βCA4*, *FBA*, and *SBP* genes of *C. sativus* and *A. thaliana* were distributed in the same group with high bootstrap values and consensus structure and were deemed to be closely related based on the phylogeny and structure analysis.

**Table 1:** The number of βCA, SBP and FBA family members in the selected plant species.

Plant species	βCA	SBP	FBA
*C. reinhardtii*	5	2	4
*P. patens*	6	3	10
*A. trichopoda*	2	1	4
*B. distachyon*	3	1	4
*O. sativa*	2	1	7
*S. bicolor*	5	1	6
*A. coerulea*	2	1	6
*G. max*	10	3	14
*B. rapa*,	13	3	13
*A. thaliana*	6	1	8
*C. sativus*	3	1	5

Based on the phylogenetic analysis, *Csa3G836520* was named as *CsβCA1* because of its the orthologue of *AtβCA1*, and *Csa5G601560* was termed as *CaβCA4*. *Csa2G252020* and *Csa5G198220* were designed as *CsFBA* and *CsSBP* because of the orthologue pairs. Primers were designed based on predicted sequences in annotated cucumber genome to obtain full-length cDNA sequence of target genes in cucumber. The PCR results and sequencing analysis proved the presence of the four target genes in cucumber genome. In this research, we focused on *CsβCA1*, *CsβCA4*, *CsSBP*, and *CsFBA1* genes to improve the photosynthesis of *C. stativus*.

### Expression patterns of cucumber *CsβCA1, CsβCA4, CsSBP*, and *CsFBA* genes

**Figure 2:**
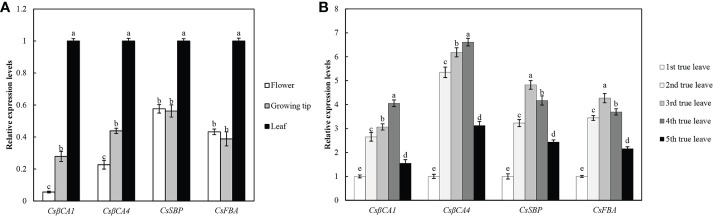
Expression patterns of target genes in different tissues in cucumber. **(A)** Tissue-specific expression profiles of four genes in different tissues, including leaf, flower, and growing tip tissue. The relative expression level in flower and growing tip were determined with the expression level of leave as internal standards to normalize. **(B)** Expression profiles of four genes in five full expanded true leaves. Small letters represent significant differences (*P*< 0.05). Labels in the figures and tables below are the same.

We performed qRT-PCR analysis of four candidate genes in different organs of cucumber under normal conditions to further investigate the expression pattern of *CsβCA1*, *CsβCA4*, *CsSBP*, and *CsFBA* genes in cucumber (**Figure 2A**). Our results showed that the expression patterns of four genes differed in different organs but exhibited similar expression patterns in the same organ. Moreover, the highest expression levels of the four genes were detected in the leaves. *CsβCA1* and *CsβCA4* were expressed at very low levels in the flowers, and the expression level of the *CsSBP* and *CsFBA* genes in the growing tips was lower than that in the flowers. These results indicated that these candidate genes played important roles in leaf growth and development. We also explored the spatial and temporal expression pattern of four candidate genes in the leaves at different stages (the first functional leaf to the fifth functional leaf) using qRT-PCR(**Figure 2B**). The expression levels of all four genes (*C*s*βCA1*, *CsβCA4*, *CsSBP*, and *CsFBA*)were increased from the first functional leaf and decreased in the fifth functional leaf. These results confirmed the different expression patterns of the four genes in the different tissues and in the leaves during cucumber development.

### Generation and identification of transgenic cucumber plants

**Figure 3:**
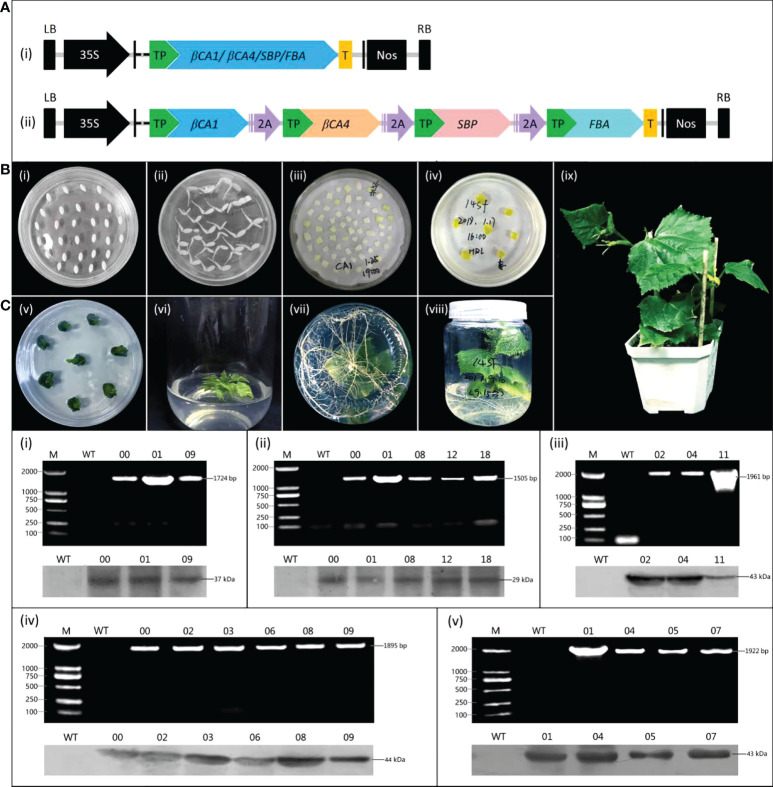
Generation and identification of transgenic plants. **(A)** Schematic diagram of vectors for cucumber transformation. (i) Separate expression vectors. (ii) The multigene expression vector. 2A: 2A linker peptide. TP: target peptide. T: flag protein tag. **(B)** The construction of cucumber transformation. (i) Cucumber seeds, (ii) Seed germination, (iii) *Agrobacterium* infection, (iv) Elimination of *Agrobacterium*, (v) Bud regeneration, (vi) Root induction, (vii) Roots, (viii) Regeneration of seedling, (ix) Seedling. **(C)** Characterization of cucumber transgenic lines, (i) *CsβCA1*, (ii) *CsβCA4*, (iii) *14SF* (Four multigene overexpression transgenic cucumber plants), (iv) *CsSBP*, or (v) *CsFBA*, by PCR and Western blotting. The upper parts are the results of PCR detection, and the lower part are the results of Western blotting. M: DL2000 DNA marker. WT: the wild-type cucumber leaves. Different numbers represent plants from representative transgenic lines.

Five corresponding expression vectors were constructed using the pCambia1305.1 vector to further investigate the effects of increasing *CsβCA1*, *CsβCA4*, *CsFBA*, and *CsSBP* expression levels on cucumbers (**Figure 3A**). Transgenic cucumber plants overexpressing target genes were generated using the *Agrobacterium*-mediated transformation method (**Figure 3B**). A pair of primers (forward primer from the 35S promoter and reverse primer from the target genes) was used to verify the presence of the target transgenic using qRT-PCR. Finally, we obtained three transgenic cucumber lines (CA1-00, CA1-01, and CA1-09) overexpressing the *CsβCA1*gene, five transgenic cucumber lines (CA4-00, CA4-01, CA4-08, CA4-12, and CA4-18) overexpressing the *CsβCA4* gene, four transgenic cucumber lines (FBA-01, FBA-04, FBA-05, and FBA-07) overexpressing the *CsFBA* gene, six transgenic cucumber lines overexpressing the *CsSBP*gene, and three transgenic cucumber lines simultaneously overexpressing four genes (**Figure 3B**). The Western blot analysis results showed that the WT plants did not show a positive signal (**Figure 3C**). By contrast, the transgenic plants exhibited obvious positive signals. Therefore, these genes were highly expressed in the transgenic cucumber lines.

### Expression level of four genes in transgenic cucumber plants

**Figure 4:**
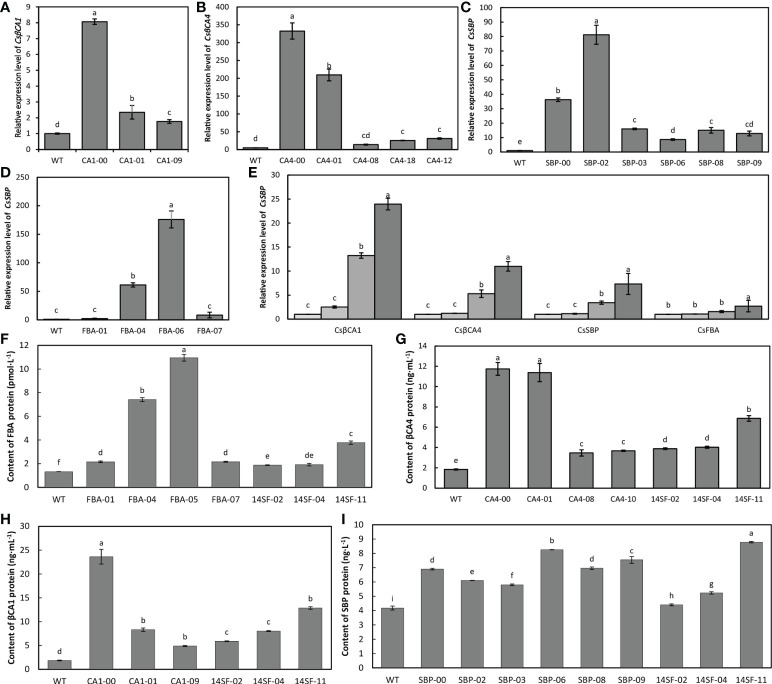
Expression analysis of transgenic lines. **(A–D)** qRT-PCR analysis of target gene transcript abundance in WT and transgenic cucumber lines. **(E)** qRT-PCR analysis of four genes in WT and 14SF lines. **(F–I)** ELISA analysis of target gene coding protein content of CsβCA1 **(F)**, CsβCA4 **(G)**, CsSBP **(H)**, and CsFBA **(I)** in different transgenic lines. Small letters in each figure represent significant differences among samples by Student’s t-test (P < 0.05).

We further investigated the expression patterns of target genes and measured the target gene-encoded protein content in the transgenic cucumber plants using qRT-PCR and ELISA. Our results showed that the expression levels of the *CsβCA1*genes were increased by 8.1 fold, 2.3 fold, and 1.7 fold in the three transgenic cucumber lines (CA1-00,01, and 09) compared with that of the WT (**Figure 4A**). Compared with the expression level of the WT plants, that of *CsβCA4* was significantly increased by 208.5 fold and 331.5 fold in the CA4-00 and CA4-01 transgenic cucumber lines; however, they only increased by 11.7 fold, 29.2 fold, and 24.6 fold in CA4-08, CA4-12, and CA4-18, respectively (**Figure 4B**). The expression level of the *CsFBA* gene in the transgenic FBA-05 lines was more than 176 fold than that of the WT plants, and the expression of the *CsFBA* gene was 2.2, 8.3, and 60.1 fold than that of the WT plants (**Figure 4D**). The *CsSBP* transcripts were significantly increased in various transgenic cucumber lines, and the highest expression level was observed in the SBP-02 lines (**Figure 4C**). We further checked the expression level of four genes in the transgenic cucumber lines overexpressing four genes and found that the expression level of four genes was significantly increased in the transgenic cucumber lines compared with the WT plants (**Figure 4D**). The protein contents of CsβCA1, CsβCA4, CsSBP, and CsFBA in the leaves of the transgenic cucumber lines also were significantly higher than those in the WT plants (**Figures 4F–I**).

### Overexpression of target genes increased the activity of RuBisCO carboxylase in the cucumber leaves

**Figure 5:**
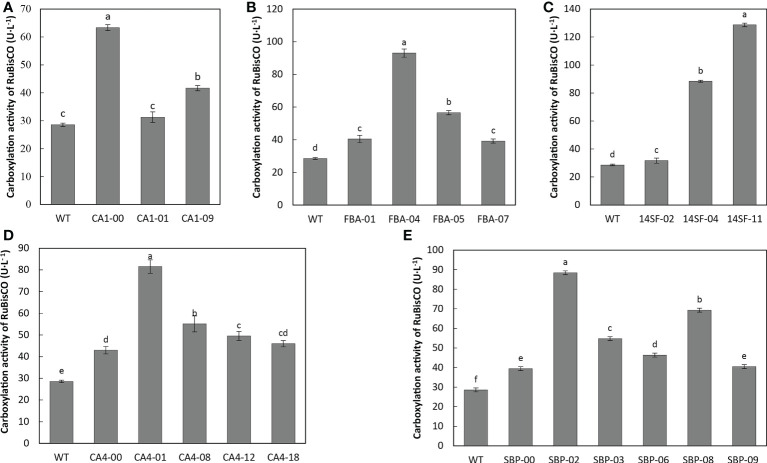
Overexpression of indicated genes increase the RuBisCO carboxylase activities in transgenic cucumber leaves. Overexpression of *CsβCA1* **(A)** *, CsFBA* **(B)**, *CsβCA4* **(D)**, or *CsSBP* **(E)** increased the RuBisCO carboxylase activities in transgenic cucumber leaves. **(C)** A multigene combination *14SF* transgenic lines showed the increased RuBisCO carboxylase activities in transgenic cucumber leaves. Small letters in each figure represent significant differences among samples by Student’s t-test (P < 0.05).

Previous studies have revealed that four genes (*CsβCA1*, *CsβCA4*, *CsSBP*, and *CsFBA*) play important roles in improving the photosynthesis capacity of plants. We explored the potential effects of these genes on the photosynthetic capacity of transgenic cucumber lines. The RuBisCO carboxylase activity in various transgenic cucumber lines was measured under atmospheric conditions before the transgenic plants transferred into low-CO_2_ conditions. Compared with the carboxylase activities of the WT plants, those of RuBisCO were significantly increased in most of the transgenic cucumber lines (**Figure 5**). The carboxylase activities of RuBisCO were increased by 2.2 fold in the transgenic cucumber lines overexpressing the *CsCA1* gene (**Figure 5A**), 2.9 fold in the transgenic cucumber lines overexpressing *CsβCA4*, and 3.3 fold in transgenic cucumber lines overexpressing *CsFBA* (**Figure 5B**).The RuBisCO carboxylase activity in the transgenic cucumber lines overexpressing four genes was significantly higher than that in the WT plants, and the highest level was observed in the 14SF-11 line (**Figure 5C**). The activity was 3.1 fold in transgenic cucumber lines overexpressing *CsSBP* (**Figure 5E**). Overall, these data suggested that the overexpression of indicated genes increased the RuBisCO carboxylase activity under low-CO_2_ conditions.

### Overexpression of target genes enhanced the photosynthesis of cucumber leaves under low-CO_2_ environment

**Figure 6:**
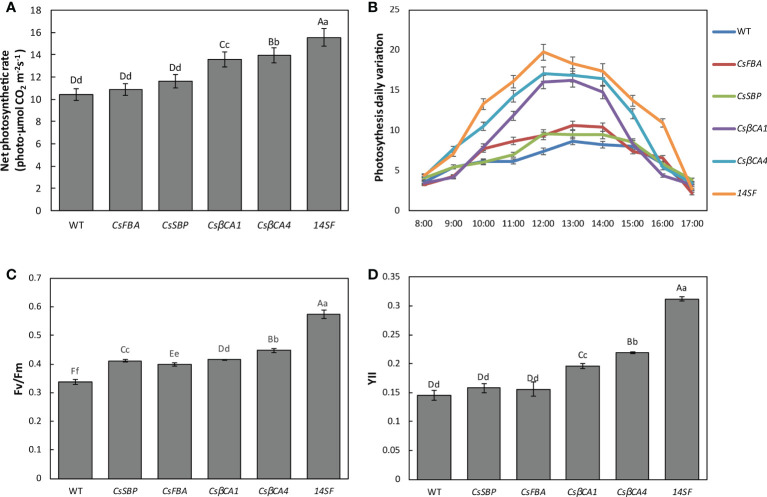
Comparison of photosynthetic and rate of WT and transgenic cucumber leaves. The net photosynthesis rate **(A)**, net photosynthesis rate dynamics in a time-course manner **(B)**, Fv/Fm **(C)** and YII **(D)** were determined. Values represent the means ± SD (n = 3) of three plants per line. Small letters in each figure represent significant differences among samples by Student’s t-test (P < 0.05).

RuBisCOis the main CO_2_-fixing enzyme of photosynthetic organisms. Improved RuBisCO carboxylase activity can enhance CO_2_ fixation. Thus, we explored the photosynthetic capacity of transgenic cucumber plants overexpressing target genes in a low-CO_2_ environment. The third functional leaves were used to measure the photosynthetic and chlorophyll fluorescence properties of the transgenic cucumber plants. Compared with the WT plants, transgenic lines overexpressing *CsβCA1* or *CsβCA4* showed significant increases in net photosynthetic rate (Pn), maximal quantum efficiency of photosystem II (PSII) photochemistry (Fv/Fm), and actual photochemical efficiency of PS II (YII) (**Figure 6**); the highest contents of Pn, Fv/Fm, and YII were detected in the transgenic cucumber lines overexpressing four genes (**Figures 6A, C, D**). The content of Pn, Fv/Fm, and YII did not differ obviously between the transgenic cucumber lines overexpressing the *FBA* and *SBP* genes and the WT plants. We also measured the diurnal change curve of photosynthesis in a single day (**Figure 6B**). Our results showed that the overexpression of Cs*βCA1* and Cs*βCA4* in transgenic cucumber resulted in greater performance in the diurnal change curve of photosynthesis than the WT and transgenic cucumber plants overexpressing the *CsFBA* and *CsSBP* genes, which did not differ significantly. Interestingly, the transgenic lines overexpressing four genes exhibited the best performance in diurnal change curve of photosynthesis among all the tested transgenic lines. Therefore, the four target genes could be modified for improving the photosynthetic capacity of cucumber under low-CO_2_ environments.

### Overexpression of certain genes promoted cucumber plant growth under low-CO_2_ environment

**Figure 7:**
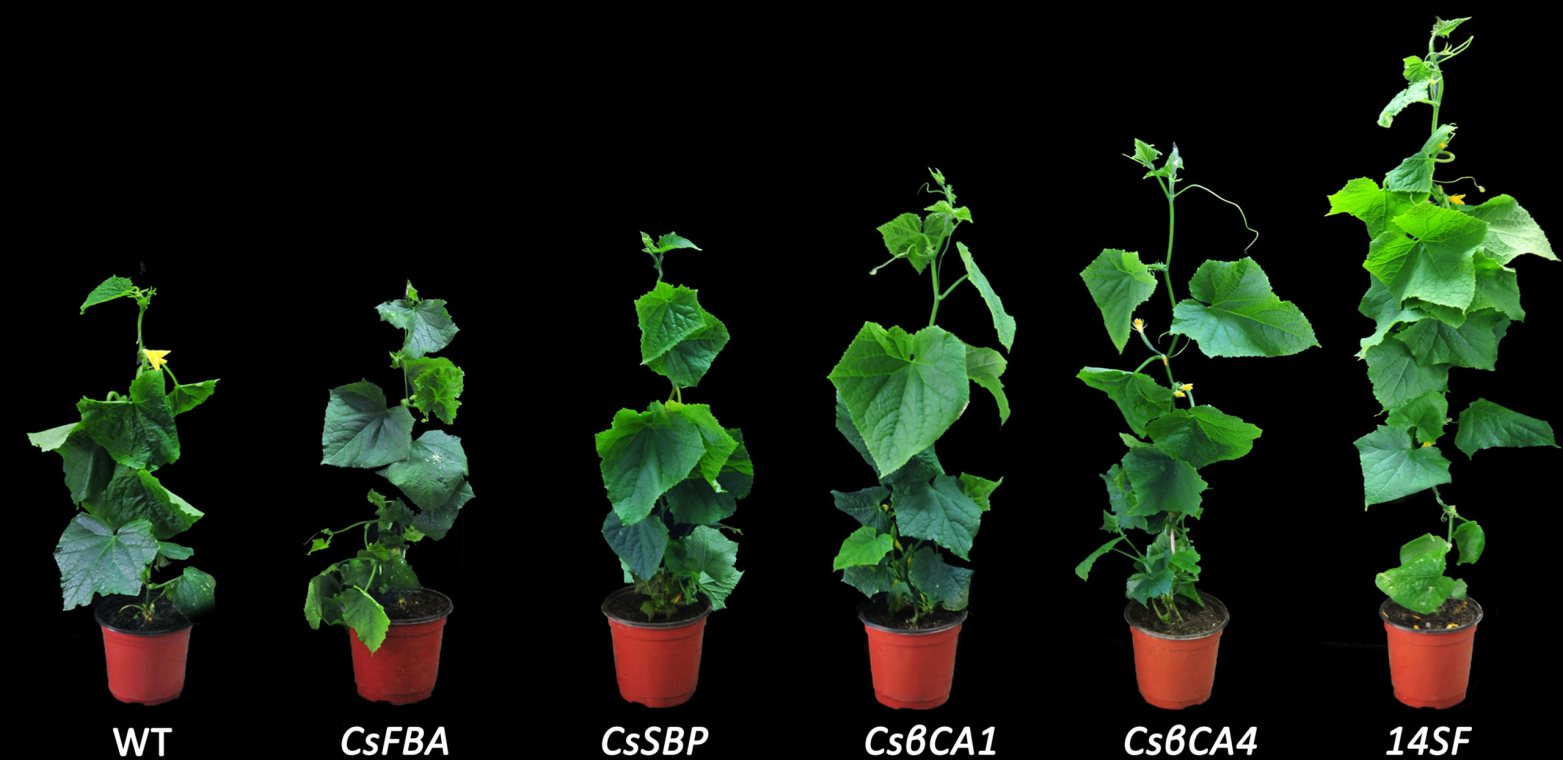
Morphological comparison of different transgenic cucumber lines after 42 days-growth in low-CO_2_ concentrations.

We compared and analyzed the growth parameters in transgenic cucumbers under low-CO_2_ environment (**Table 2** and **Figure 7**). The heights of the transgenic cucumber plants overexpressing *CsβCA1* or *CsβCA4* were significantly higher than that of the WT plants under low-CO_2_ environment. Furthermore, no remarkable differences were observed in the transgenic lines overexpressing *CsSBP* and *CsFBA* compared with the WT plants. The stem diameters were significantly increased in the *CsβCA1* overexpressing transgenic plants. No obvious difference was found in the left lengths or areas between the transgenic lines overexpressing individual genes and the WT plants. The plant heights, stem diameters, left lengths, and widths of the transgenic cucumber plants overexpressing four genes were significantly increased compared with those of the WT plants (**Table 2**).

**Table 2:** Comparison of the growth parameters between overexpression lines and the control plants.

	Plant height: cm	Stem diameter: mm	Leaf length:cm	Leaf width: cm	Dry weight of aboveground: g	Dry weight of underground: g	Weight of single fruit: g
WT	68.1 ± 1.9 d	8.3 ± 0.1 d	9.1 ± 0.1 b	10.4 ± 0.3 b	21.1 ± 3.0 c	1.3 ± 0.4 c	539.9 ± 5.8 d
*CsFBA*	70.7 ± 1.2 cd	8.5 ± 0.3 d	9.3 ± 0.5 b	11.0 ± 0.5 b	23.4 ± 1.2 bc	1.6 ± 0.1 c	569.5 ± 14.2 cd
*CsSBP*	76.2 ± 4.5 bc	8.9 ± 0.3 d	9.8 ± 0.2 b	11.9 ± 1.0 b	23.5 ± 2.5 bc	1.5 ± 0.2 c	586.0 ± 1.9 bc
*CsβCA4*	80.9 ± 2.7 b	9.9 ± 0.5 c	10.7 ± 0.1 b	12.3 ± 0.3 b	27.1 ± 2.1b	1.6 ± 0. 5 b	625.7 ± 18.6 b
*CsβCA1*	82.8 ± 1.4 b	11.3 ± 0.7 b	10.7 ± 0.1 b	12.7 ± 0.2 b	29.5 ± 1.2 b	1.8 ± 0.3 b	625.0 ± 60.5 b
*14SF*	103.3 ± 4.7 a	13.1 ± 0.2a	15.7 ± 1.7 a	20.0 ± 2.1 a	36.0 ± 6.0 a	2.5 ± 0.1 a	736.1 ± 23.0 a

Small letters a, b, c, and d represent significant differences among samples by Student’s t-test (P < 0.05).

Values represent the means ± SD (n = 3) of three plants per line.

We also analyzed the single-fruit weight and dry weight of the cucumber plants. We found that all single-gene transgenic lines showed similar aboveground and belowground dry weights (**Table 2**) to the control plants, except for the *CsβCA1*-transgenic plants, which showed a significantly higher aboveground dry weight. Meanwhile, the 14SF line showed increased aboveground and belowground dry weights compared with the WT control. The single-gene overexpression lines showed similar single-fruit weights, except for the *CsFBA* transgenic plants, which displayed a low fruit weight. Similarly, the 14SF lines revealed the highest single-fruit weight.

## Discussion

Photosynthesis is the primary determinant of crop productivity, and any gain in photosynthetic CO_2_ assimilation per unit of leaf area has the potential to increase yield (Faralli and Lawson, 2020). CO_2_ is an important element in photosynthesis, which is a process that converts energy from sunlight to chemical energy stored in glucose. A large proportion of the limitation of carbon assimilation in plants by the C3 cycle is due to the catalytic property of the enzyme RuBisCO (Raines, 2022). Genetic engineering, including gene editing and transgenic methods, has proven to be an effective approach to improve the photosynthetic capacity of crops. Chen et al. (2019) introduced exogenous glycolate catabolic pathway into cucumber and obtained transgenic plants that can exhibit high photosynthetic efficiency under low-CO_2_ environment. De Souza et al. (2022) overexpressed *VPZ* gene in soybean, which effectively improved the efficiency of photosynthesis. Specifically, it resulted in a 33% increase in soybean yield (De Souza et al., 2022). However, with overexpression of *SBP* or *FBA* in plants, the improvements in photosynthesis and biomass yield occurred only under elevated CO_2_ conditions (Rosenthal et al., 2011; Uematsu et al., 2012), such improvements are significant in greenhouse environments in winter (Rosenthal et al., 2011), the authors of these studies attribute the results to the shorter day length and the lower light level.

βCAs are important enzymes involved in plant carbon transport and carbon concentrating mechanism (Polishchuk, 2021) and play an important role in maintaining crop photosynthesis under low-CO_2_ conditions (DiMario et al., 2016). Studies of *βCA* mutants with altered *βCA* gene expression in *Arabidopsis* have confirmed that *βCAs* are critical for photosynthesis, especially under low-CO_2_ conditions. Similar to *βCAs*, *ictB* are also involved in carbon transport and carbon concentrating mechanism in *Cyanobacteria*. The introduction of the *inorganic CO_2_ transporter B* (*ictB*) together with the overexpression of *SBP* and *FBA* in transgenic tobacco resulted in an increase in photosynthesis and biomass (Simkin et al., 2015). Inspired by this strategy, we overexpressed *CsβCA1*, *CsβCA4*, *CsSBP*, and *CsFBA* separately or co-overexpressed the four genes in cucumber to verify the feasibility of manipulation of multiple target genes for improving cucumber photosynthesis and biomass yield under low-CO_2_ conditions.

**Figure 8:**
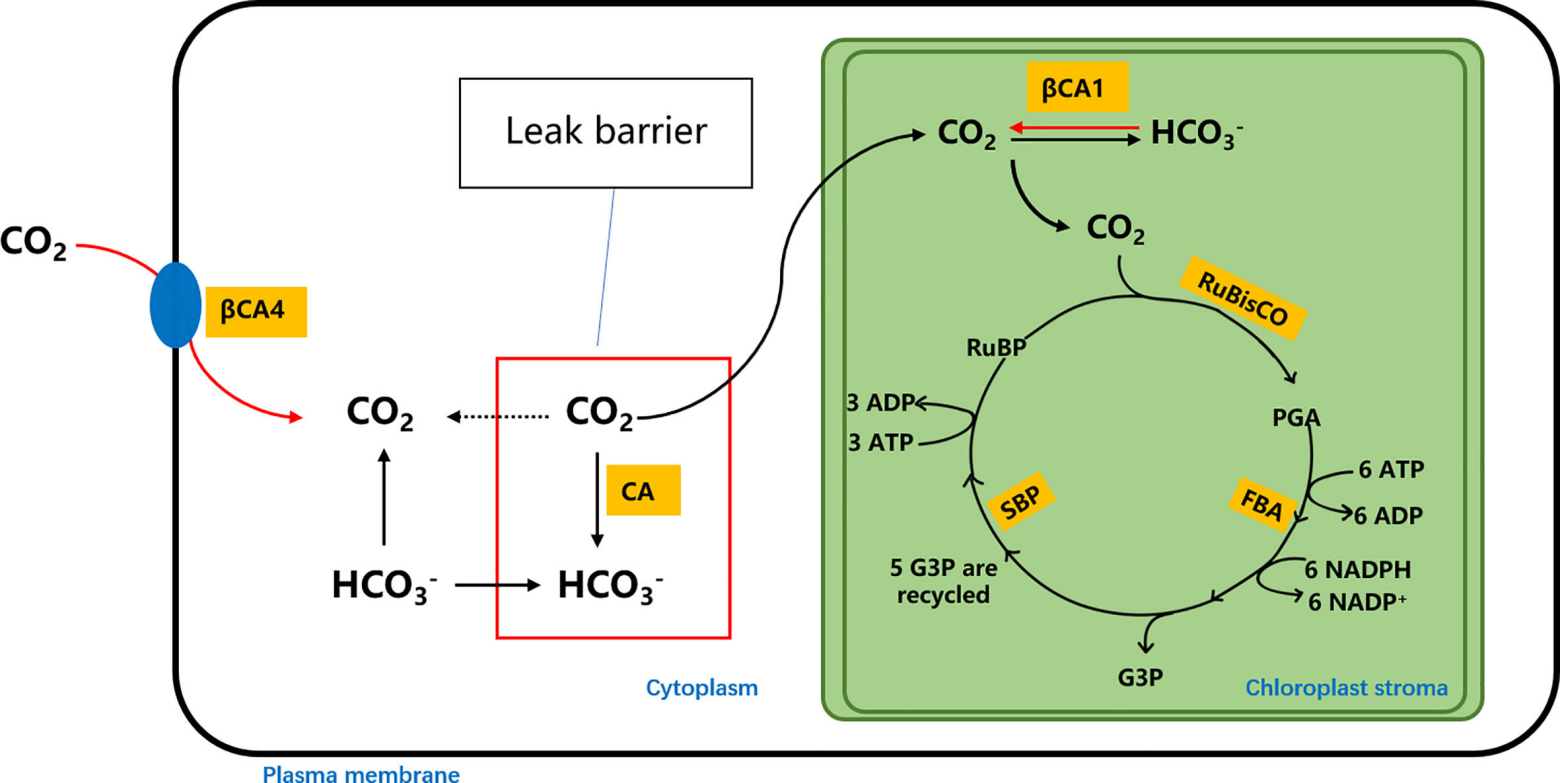
Superposition of plant photosynthetic carbon assimilation pathway. The highlighted part represents the locations of βCA1, βCA4, SBP and FBA enzymes.

Our results showed that the overexpression of individual target genes (*CsβCA1*, *CsβCA4*, *CsSBP*, or *CsFBA*) increased the RuBisCO activity under the atmospheric CO_2_ environment, which is consistent with previous studies. Moreover, transgenic cucumber overexpressing the *CsβCA*1 and *CsβCA4* genes exhibited significantly increased photosynthesis and biomass yield under a low-CO_2_ environment. Meanwhile, the overexpression of the *CsSBP* and *CsFBA* genes in transgenic cucumber resulted in no significant difference in photosynthesis and biomass yield when compared with the WT plants. Interestingly, our further analysis of the chlorophyll fluorescence parameters in transgenic cucumber under low-CO_2_ environment revealed that the maximum photochemical efficiency of PSII in transgenic cucumber plants overexpressing individual four target genes was significantly higher than that of the WT plants, which implies that the *CsβCA*, *CsSBP*, and *CsFBA* genes played different roles in regulating photosynthesis. *CsβCA* genes are involved mainly in CO_2_ fixation, and *CsSBP* and *CsFBA* genes are involved mainly in the RuBP regeneration phase of the C3 cycle, which serves to provide a substrate for carbon assimilation (**Figure 8**). Previous studies have proven that increasing the activity of SBP and FBA can accelerate the regeneration of RuBP and enhance the carbon assimilation rate of plants under sufficient CO_2_. However, in near-airtight solar greenhouses, CO_2_ concentrations may drop to very low levels in autumn, winter, and spring and cannot be replenished in time. Long-term CO_2_ deficiency is the main limiting factor for photosynthetic carbon assimilation. Thus, the *CsFBA* and *CsSBP* overexpressing transgenic plants did not exhibit high efficacy, which suggests that a low-CO_2_ concentration inhibits the activity of SBP and FBA.

In this study, the transgenic cucumber plants overexpressing four target genes were generated for the first time. Our results showed that transgenic cucumber plants overexpressing four target genes simultaneously displayed better performance, including photosynthesis, chlorophyll fluorescence parameters, biomass, and growth and development, under a low-CO_2_ environment in comparison with transgenic cucumber plants overexpressing individual target genes and WT plants. The increased activity of βCA promoted the decomposition of HCO_3_^−^ into CO_2_, increased the CO_2_ concentration around RuBisCO, and maintained the carboxylation activity of RuBisCO, which improved the ability of cucumber to utilize low-CO_2_. The increased RuBisCO carboxylate activity facilitated the rapid consumption of RuBP, and the overexpressed SBP and FBA catalyzed RuBP regeneration which provided the RuBP substrate for carbon fixation. The cumulative effect of *CsβCA1*, *CsβCA4*, *CsSBP*, and *CsFBA* accelerated the assimilation of CO_2_. Thus, 14SF transgenic plants could maintain a high photosynthetic efficiency and biomass yield even under low-CO_2_ concentration environment.

In conclusion, we confirmed the potential use of multiple enzymes of the photosynthetic carbon metabolism for improving photosynthesis and growth for cucumbers in low-CO_2_ conditions. The overexpression of *CsβCA* genes in cucumber could improve the photosynthetic capacity of cucumber leaves and increase the biomass yield of cucumber under low-CO_2_ conditions. Meanwhile, the genetic manipulation of multiple enzymes of CO_2_ assimilation, such as *CsβCA*, *CsSBP*, and *CsFBA*, showed potential application for achieving photosynthesis and growth improvement goals for greenhouse cucumber plants.

## Data availability statement

The datasets presented in this study can be found in online repositories. The names of the repository/repositories and accession number(s) can be found in the article/**Supplementary Material**.

## Author contributions

Z-FC, T-HW, G-MX, and G-FT carried out this experiment and collected the manuscript data. Z-FC, T-HW, G-MX, SS, and G-FT wrote the manuscript. Z-FC, T-HW, C-YF, X-XG, T-LZ, H-FG, W-ZL, G-MX, and G-FT approved the final manuscript. All authors have read and approved the final manuscript.

## Funding

This study is financially supported by Guizhou Province Science and Technology Plan Project (QKHZC[2021] No.207), Guizhou Province Youth Science and Technology Top Talent Project (QJJ[2022] No.89), and Guizhou Vegetable Modern Agricultural Industry Technology System (GZCYTX2022-01).

## Conflict of interest

The authors declare that the research was conducted in the absence of any commercial or financial relationships that could be construed as a potential conflict of interest.

## Publisher’s note

All claims expressed in this article are solely those of the authors and do not necessarily represent those of their affiliated organizations, or those of the publisher, the editors and the reviewers. Any product that may be evaluated in this article, or claim that may be made by its manufacturer, is not guaranteed or endorsed by the publisher.
