# Supplementary material for: Multigene manipulation of photosynthetic carbon metabolism enhances the photosynthetic capacity and biomass yield of cucumber under low-CO2 environment
Source: Front Plant Sci. 2022 Oct 18;13:1005261. doi: 10.3389/fpls.2022.1005261 (PMC9623318; doi:10.3389/fpls.2022.1005261)
Supplement: Supplementary file 1 [file DataSheet_1.pdf]

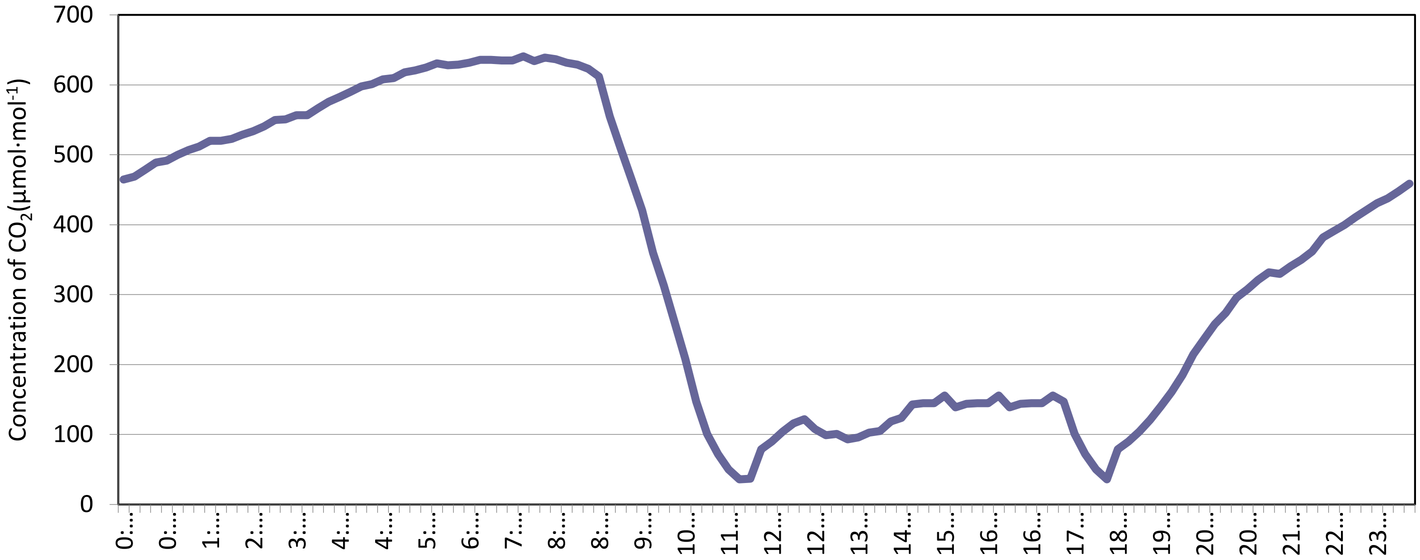

FIGURE S1 Daily variation curve of CO_2 concentration in an enclosed solar greenhouse for cucumber cultivation

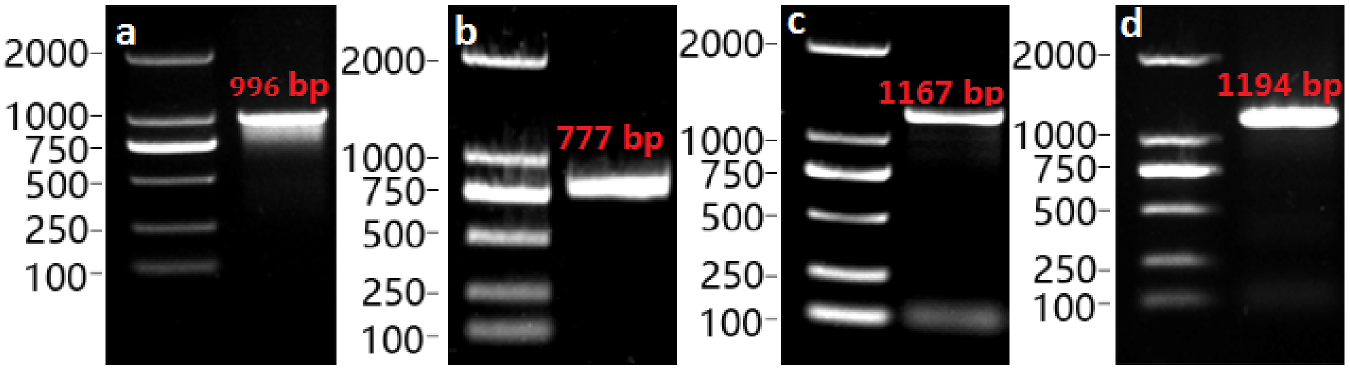

FIGURE S2 PCR amplification for gene coding regions of *βCA1*(a), *βCA4*(b), *FBA*(c) and *SBP*(d) genes in cucumber.

Table S1 Primer and antigen sequences used in this study.

Name	Sequences	Function
**βCA1-F**	ATGTCGACGGCTTCCATTA	Gene clone
**βCA1-R**	TCATACAGACAAAGGCTTAGTAACGT	Gene clone
**βCA4-F**	ATGGCGCAAGAGTCCTACG	Gene clone
**βCA4-R**	TCAAACAGCTAGAGAAGGTGAAA	Gene clone
**SBP-F**	ATGGAGACTGGAATTGCTTG	Gene clone
**SBP-R**	TTAAGCTGCAGCACCAACAGG	Gene clone
**FBA-F**	ATGGCTTCTGCTTCTCTTC	Gene clone
**FBA-R**	TTAGTATGTGTAGCCCTTGACG	Gene clone
**35S-F**	CATGGAGTCAAAGATTCAAATAGAG	expression
**35S-R**	AGTCCCCCGTGTTCTCTCC	expression
**CA1+CA4-F2m**	CTAAGCCTTTGTCTGTAATGGCGCAAGAGTCCTACG	SEO PCR
**CA1+CA4-R1m**	TCGTAGGACTCTTGCGCCATTCAACAGACAAAGGCTTAGTAACGT	SEO PCR
**SBP+FBA-F2m**	CTGTTGGTGCTGCAGCTATGGCTTCTGCTTCTCTTC	SEO PCR
**SBP+FBA-R1m**	GAAGAGAAGCAGAAGCCAAGCTGCAGCACCAACAGG	SEO PCR
**tua-F**	ACGCTGTTGGTGGTGGTAC	qRT-PCR
**tua-R**	GAGAGGGGTAAACAGTGAATC	qRT-PCR
**Q-CA1-F**	CACCAAGCCCAAGAAGATC	qRT-PCR
**Q-CA1-R**	GTTGCCTTCAAATCTCCCT	qRT-PCR
**Q-CA4-F**	ATTGCCAACATGGTTCCAC	qRT-PCR
**Q-CA4-R**	AAACATTCACAGCCTCCTT	qRT-PCR
**Q-SBP-F**	TTTTAGTGTTGCATTTGACCCAC	qRT-PCR
**Q-SBP-R**	ATGTTGCCATTTGCCTTCG	qRT-PCR
**Q-FBA-F**	GGGTCTTGCTCGCTACGCT	qRT-PCR
**Q-FBA-R**	AGGGCTTGCTTTGTCTTTGCT	qRT-PCR
**CA1**	RLSNNNTPSPSF	ELISA
**CA4**	PPFDKTKYSG	ELISA
**SBP**	IENLDERTQVA	ELISA
**FBA**	EWVKGQSLRQPSV	ELISA
**RuBisCO**	PQTETKASVGFKAGVKDYKLTYYTPDYETKDTDILAAFRVTPQPGVPPEEAGAAVA<br>AESSTGTWTTVWTDGLTSLDRYKGRCYGLEPVAGEENQYIAYVAYPLDLFEEGSVT<br>NMFTSIVGNVFGFKALRALRLEDLRIPTAYIKTFQGPPHGIQVERDKLNKYGRPLLG<br>CTIKPKLGLSAKNYGRAVYECLRGGLDFTKDDENVNSQPFMRWRDRFLFCAEAIF<br>KSQAETGEIKGHYLNATAGTCEEMMKRAVFARELGVPIVMHDYLTGGFTANTSLA<br>HYCRDNGLLLHIHRAMHAVIDRQKNHGMHFRVLAKALRLSGGDHVHAGTVVGK<br>LEGEREITLGFVDLLRDDFVEKDRSRGIYFTQDWVSLPGVLPVASGGIHVWHMPA<br>LTEIFGDDSVLQFGGGTLGHPWGNAPGAVANRVALEACVQARNEGRDLAREGN<br>EIIREASKWSPELAAACEVWKEIKFEFEAMDTL	ELISA
**Flag Taq**	DYKDDDDK	Western Blotting

Table S2 Target peptide and linker peptide sequences

SSU protein sequence
MASSMLSSATMVASPAQATMVAPFNGLKSSAAFPATRKANNDITSITSNGGR
VNCMQVWPPIGKKKFETLSYLPDLTDS
SSU DNA sequence
ATGGCTTCCTCTATGCTCTCTTCCGCTACTATGGTTGCCTCTCCGGCTCAG
GCCACTATGGTCGCTCCTTTCAACGGACTTAAGTCCTCCGCTGCCTTCCC
AGCCACCCGCAAGGCTAACAACGACATTACTTCCATCACAAGCAACGGC
GGAAGAGTTAACTGCATGCAGGTGTGGCCTCCGATTGGAAAGAAGAAGT
TTGAGACTCTCTCTTACCTTCCTGACCTTACCGATTCC
2A DNA sequence optimized with cucumber codons
CTGTTGAACTTTGACCTACTTAAGTTGGCTGGTGACGTTGAGTCTAACCT
TGGGCCT
2A amino acid sequence
QLLNFDLLKLAGDVESNPGP
