# Supplementary material for: Multigene manipulation of photosynthetic carbon metabolism enhances the photosynthetic capacity and biomass yield of cucumber under low-CO2 environment
Source: Front Plant Sci. 2022 Oct 18;13:1005261. doi: 10.3389/fpls.2022.1005261 (PMC9623318; doi:10.3389/fpls.2022.1005261)
Supplement: Supplementary file 2 [file Presentation_1.pptx]

## Slide 1
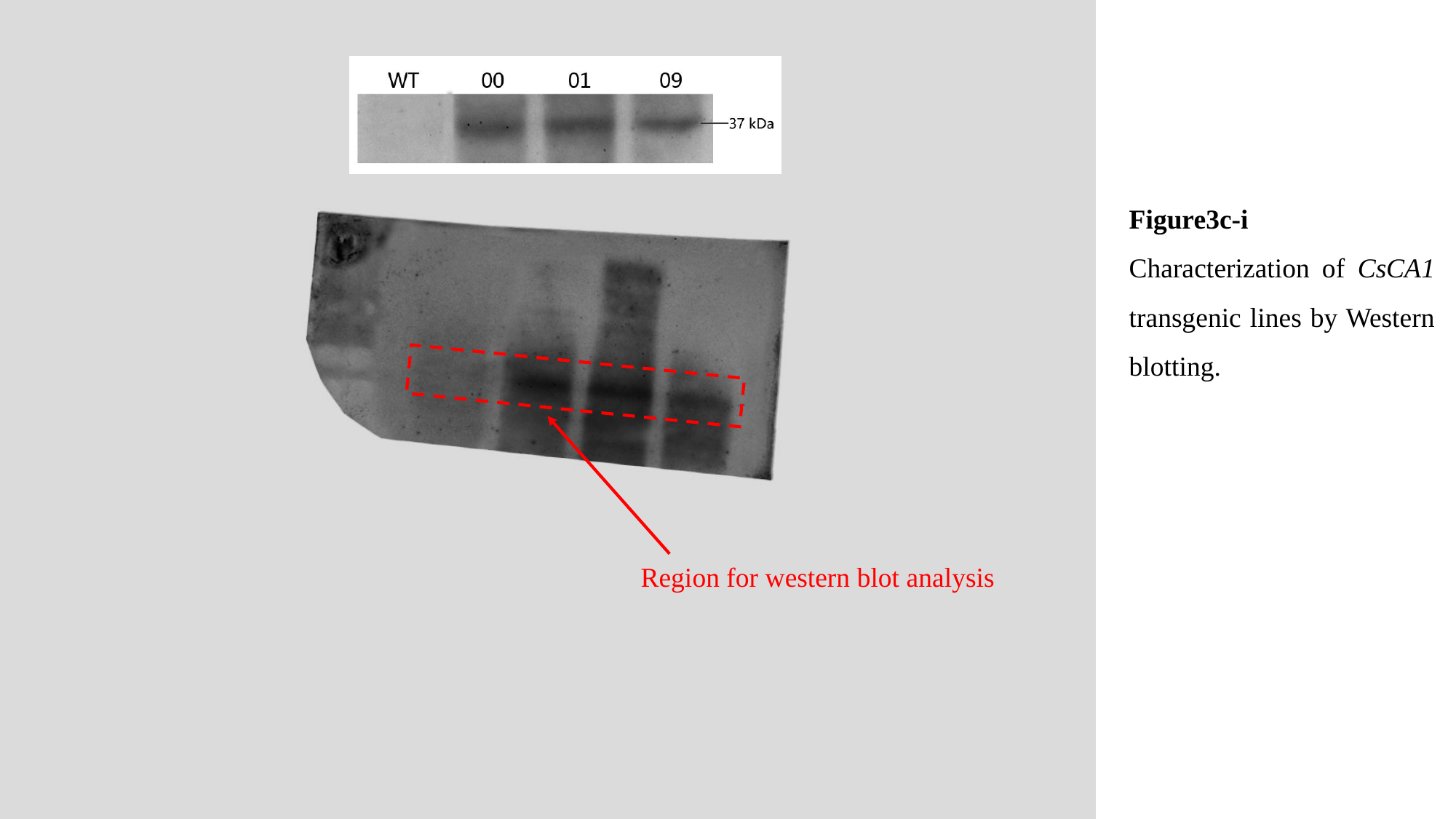

Figure3c-i Characterization of CsCA1 transgenic lines by Western blotting.
Region for western blot analysis

## Slide 2
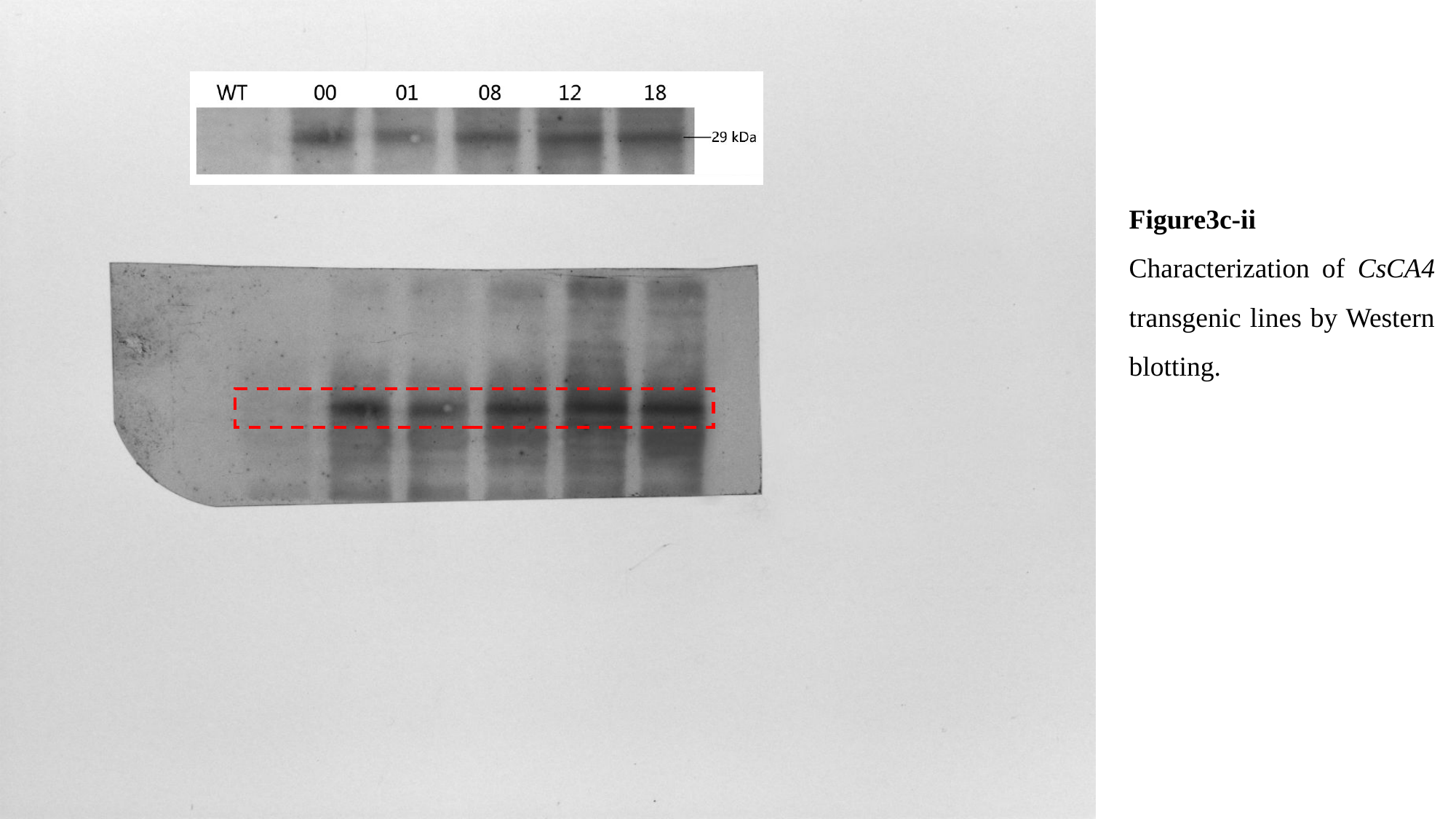

Figure3c-ii Characterization of CsCA4 transgenic lines by Western blotting.

## Slide 3
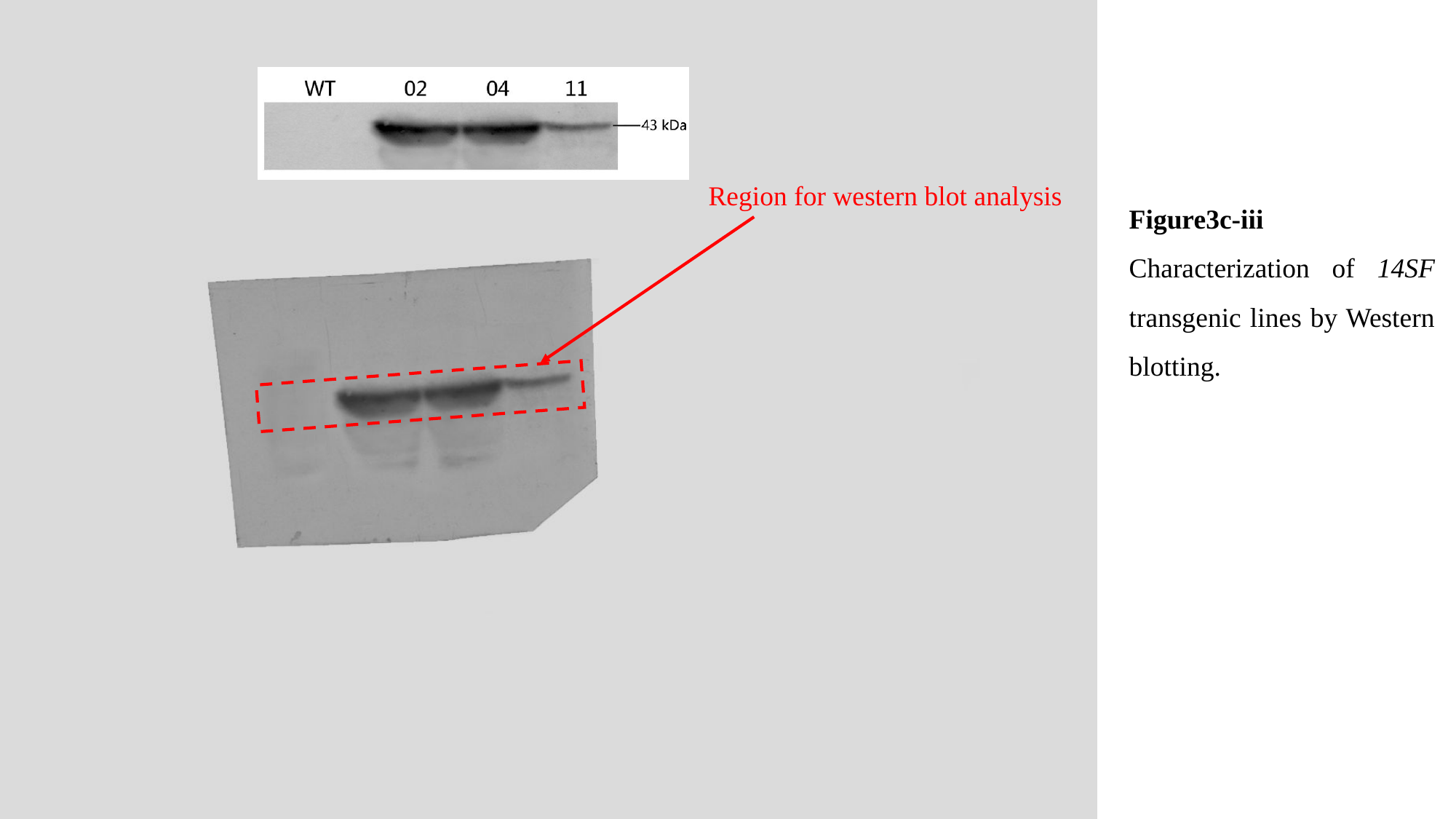

Region for western blot analysis
Figure3c-iii Characterization of 14SF transgenic lines by Western blotting.

## Slide 4
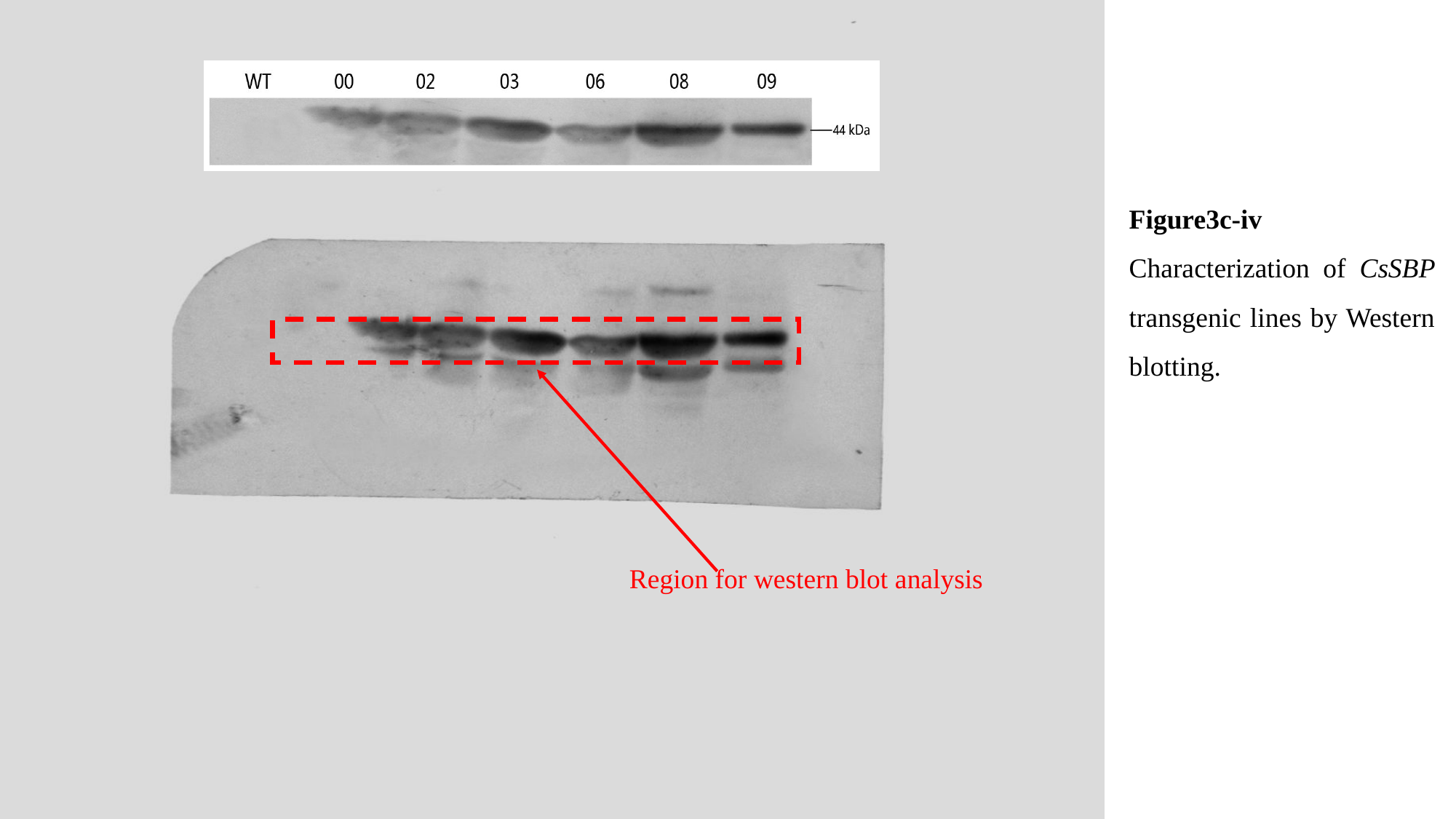

Figure3c-iv Characterization of CsSBP transgenic lines by Western blotting.
Region for western blot analysis

## Slide 5
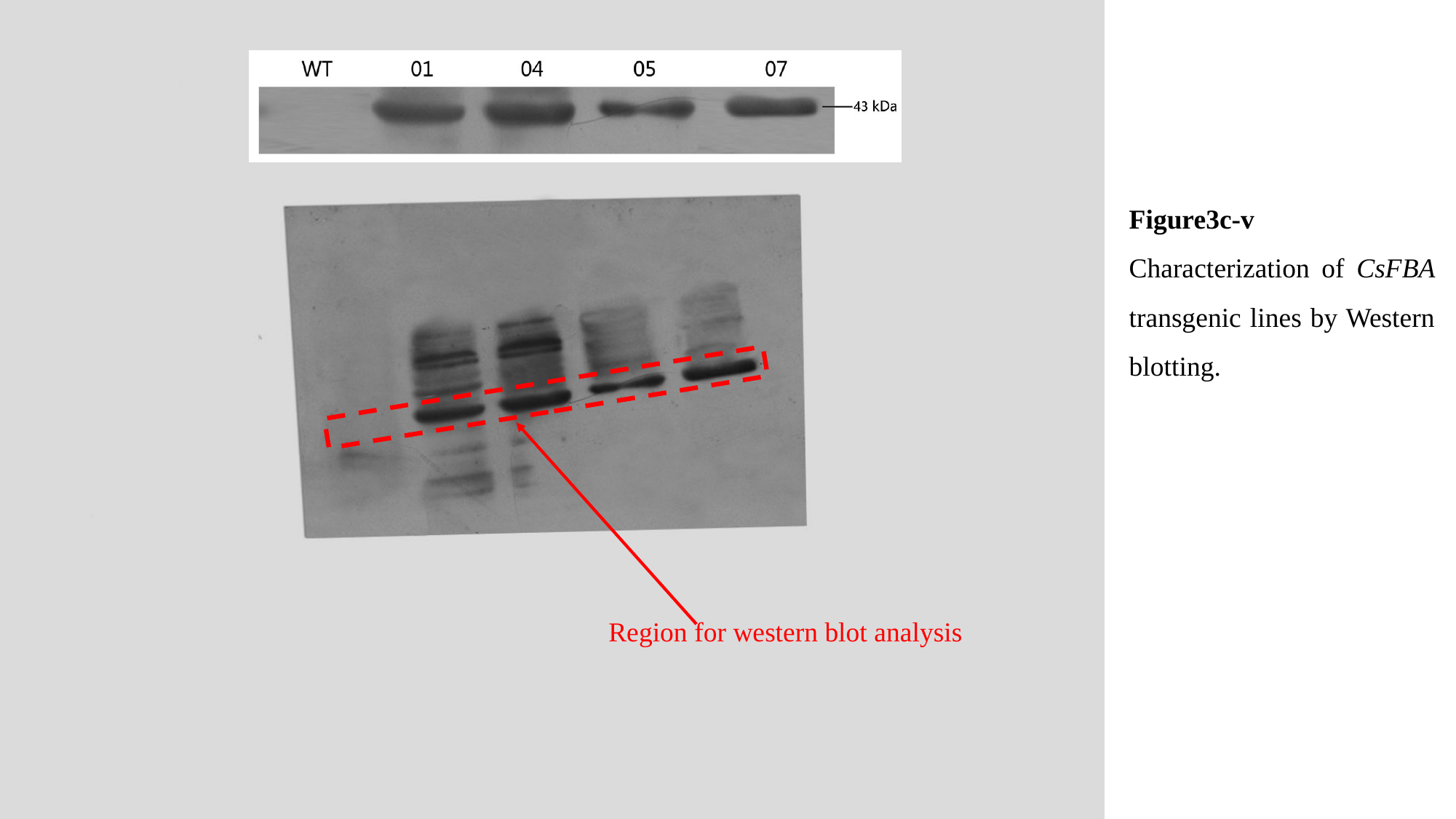

Figure3c-v Characterization of CsFBA transgenic lines by Western blotting.
Region for western blot analysis
